# Early Prediction of Lung Cancers Using Deep Saliency Capsule and Pre-Trained Deep Learning Frameworks

**DOI:** 10.3389/fonc.2022.886739

**Published:** 2022-06-17

**Authors:** Kadiyala Ramana, Madapuri Rudra Kumar, K. Sreenivasulu, Thippa Reddy Gadekallu, Surbhi Bhatia, Parul Agarwal, Sheikh Mohammad Idrees

**Affiliations:** ^1^ Department of Information Technology (IT), Chaitanya Bharathi Institute of Technology, Hyderabad, India; ^2^ Department of Computer Science and Engineering (CSE), G. Pullaiah College of Engineering and Technology, Kurnool, India; ^3^ Department of Information Technology, Vellore Institute of Technology, Vellore, India; ^4^ Department of Information Systems, College of Computer Sciences and Information Technology, King Faisal University, Al Hasa, Saudi Arabia; ^5^ Department of Computer Science and Engineering (CSE), Jamia Hamdard, India; ^6^ Department of Computer Science Institutt for datateknologi og informatikk (IDI), Norwegian University of Science and Technology, Gjøvik, Norway

**Keywords:** computer tomography (CT) scan images, saliency segmentation, pre-trained models, whale optimization, DenseNet, VGG-16, inception models

## Abstract

Lung cancer is the cellular fission of abnormal cells inside the lungs that leads to 72% of total deaths worldwide. Lung cancer are also recognized to be one of the leading causes of mortality, with a chance of survival of only 19%. Tumors can be diagnosed using a variety of procedures, including X-rays, CT scans, biopsies, and PET-CT scans. From the above techniques, Computer Tomography (CT) scan technique is considered to be one of the most powerful tools for an early diagnosis of lung cancers. Recently, machine and deep learning algorithms have picked up peak energy, and this aids in building a strong diagnosis and prediction system using CT scan images. But achieving the best performances in diagnosis still remains on the darker side of the research. To solve this problem, this paper proposes novel saliency-based capsule networks for better segmentation and employs the optimized pre-trained transfer learning for the better prediction of lung cancers from the input CT images. The integration of capsule-based saliency segmentation leads to the reduction and eventually reduces the risk of computational complexity and overfitting problem. Additionally, hyperparameters of pretrained networks are tuned by the whale optimization algorithm to improve the prediction accuracy by sacrificing the complexity. The extensive experimentation carried out using the LUNA-16 and LIDC Lung Image datasets and various performance metrics such as accuracy, precision, recall, specificity, and F1-score are evaluated and analyzed. Experimental results demonstrate that the proposed framework has achieved the peak performance of 98.5% accuracy, 99.0% precision, 98.8% recall, and 99.1% F1-score and outperformed the DenseNet, AlexNet, Resnets-50, Resnets-100, VGG-16, and Inception models.

## 1 Introduction

Lung tumor (LT) is the most lethal cancer on the planet. As a result, numerous countries are working on early detection measures for lung disease. The NLST experiment (1) found that screening high-risk participants three times a year with low-dose computed tomography (CT) reduces death rates significantly (2). As a result of these procedures, a radiologist will have to examine a large number of CT scan images. Because lesions are difficult to identify, even for qualified clinicians, the strain on radiologists grows exponentially as the quantity of CT scans to review grows.

Lung cancer is the second most prevalent cause of cancer death in people. Cancers of the bladder, breast, colon, cervix and, prostate have 5-year survival rates of over 80%. Thus, early identification of lung cancer is critical to reducing mortality or facilitating full care. Due to their thin cell layers (0.2-1*mm*) and lack of symptoms, early lung malignancies and precancers such as dysplasia and carcinoma *in situ* (CIS) are difficult to identify visually using traditional diagnostic procedures such as medical imaging. In clinical practice, roughly 80% of cases are advanced when initially diagnosed and verified, losing the best chance for surgical therapy. Clearly, early detection of lung cancer is clinically significant.

With the predicted rise in the number of preventive/early-detection measures, scientists are developing automated solutions to assist doctors in decreasing their workload, improving diagnostic precision by minimizing subjectivity, speeding up analysis, and lowering medical costs. Specific traits must be detected and assessed to identify the cancerous cells in the lung region. Cancer risk can be determined by the observed features and their combination. Even for an experienced medical expert, this work is challenging because nodule existence and a positive cancer diagnosis are not easily linked. Volume, shape, subtlety, firmness, spiculation, sphericity, and other previously described properties are used in common computer-assisted diagnostic (CAD) techniques.

**Figure 1:**
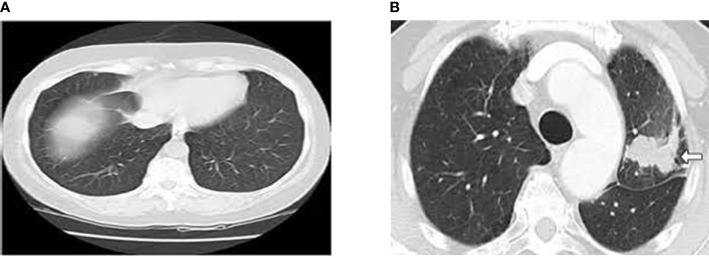
**(A)** Normal CT Lung Image **(B)** Abnormal CT Lung Image (Cancer Image).

Machine learning (ML) techniques like Support Vector Machine (SVM) are utilized to identify the nodules as benign or cancerous. Despite the fact that numerous works employ comparable machine learning frameworks (3–10), the limitation of this technique is that in order for the system to function properly, different variables must be customized, making it difficult to repeat results. Furthermore, the lack of uniformity among CT scans and screening parameters makes these systems vulnerable. The development of deep training in CAD systems might do end-to-end identification by acquiring the most essential factors during training. The network is resistant to variations since it gathers tumor features in multiple CT scans with repeated modes. By adopting a training set that is rich in variability, the system may be able to learn invariant properties from malignant nodules intrinsically and enable higher performances (11, 12). Since no characteristics are generated, the system may be able to understand the relationship between traits and disease using the data provided on its own. Once trained, the network should be able to generalize its training and recognize cancerous lesions (or malignancy at the clinical bedside) on cases reported that have never been observed before (13, 14). **Figure 1** shows the normal and abnormal CT lung images. Early classification and classification of lung cancers play a critical role in designing an intelligent and accurate diagnosis system (15). With the advent of machine and deep learning algorithms, the design of early diagnosis systems has reached new heights. Machine learning algorithms such as artificial neural networks (ANN), Support Vector Machines (SVM), Naïve Bayes Classifiers (NB), and Ensemble classifiers (EC) are primarily used for an early diagnosis of lung cancers (16). Also, deep learning is considered to be the most promising field which can enhance the performance of various medical imaging and diagnosis systems (17).

However, handling the images with different imaging protocols remains a real challenge to train the learning modes for greater performance. To compensate for the above drawback of learning models, this paper proposes the novel hybrid intelligent diagnosis framework Deep Fused Features Based Reliable Optimized Networks (DFF-RON), which fuses the saliency maps and convolutional layers for better segmentation and feature extraction that are used to train the ant-lion optimized single feedforward networks. To the best of our knowledge, this is the first work that has integrated the fused features and optimized learning networks to design an efficient and high-performance CT-based lung cancer diagnosis system.

### 1.1 Contribution of the Research Work

1. A novel hybrid deep learning based model is proposed for the early detection of lung cancer using CT scan images. The proposed architecture has been trained with LIDC datasets and performance metrics have been calculated and compared with other existing models.
2. The proposed architecture introduces the capsule network’s better segmentation and transfer learning for feature extraction. Also, the proposed fusion algorithm can increase the high diagnosis rate.
3. The whale optimization algorithm is proposed for training the features obtained from the hybrid fusion of saliency maps and capsule networks. The feed-forward layers are designed based on the principle of Extreme Learning Machines (ELM).

The rest of the paper is organized as follows: Section-II presents the related works proposed by more than one author. The working mechanism of the saliency maps, CNN layers, ant lion optimization, and feedforward networks are presented in Section-III. The dataset descriptions, experimentations, results, findings, and analysis are presented in Section-IV. Finally, the paper is concluded in Section-V with future enhancements.

## 2 Related Works

In De Bruijne (18), the presented framework looked at the most up-to-date lung cancer detection and diagnosis methods. Using standardized databases LIDC-IDRI, LUNA 16, and Super Bowl Dataset 2016, the newest lesion detection, identification, and detectors are acquainted with labeled models. According to the author Jindal et al. (19), these are the most common and typical threshold CT data considered for diagnosing. The authors in Nalepa and Kawulok (20) developed the modified-CNN in order to recognize the tumor cells in the lung regions with the segmented images. The ACM method has been used for segregating the tumor region initially and identifying cancer or normal cells.

The label-free techniques do not injure cells or cause effects on cell structure or intrinsic features. To enhance cell identification using recorded optical profiles, this study combined advancements in optical coherence tomography with Prony methods. In Ganesan et al. (21), the framework finds signature genes by improving Tobacco Exposure Pattern (TEP) Prediction model and revealing their interaction connections at many biological levels. TTZ Kasinathan et al. (22) is a new way to extract core features and use them as an input variable in the TEP classification model. With two distinct LUAD datasets used to train and evaluate the TEP classification model, 34 genes were identified as nicotine-associated mutation signature genes, with an accuracy of 94.65% for training data and 91.85% for validation data.

The researcher examined tissue samples and devised a categorization method to discriminate between five types of pulmonary and colorectal tissues (two benign and three malignant). According to the observations, the suggested approach can detect tumor cells up to 96.33% of the time (23). The framework presented in Suzuki (24) described how to use computer-assisted diagnostics to assess EGFR mutation status, including gathering, evaluating, and merging multi-type interdependence characteristics. This research uses a new hybrid network model based on CNN-RNN architecture. CNN is used to extract image quantitative properties, and the link between different types of features is modeled. Their study indicate that multi-type dependency-based feature representations beat single-type feature representations (accuracy = 75%, AUC = 0.78) when compared to conventional features extracted.

The 3D_Alex Net unsupervised learning model (25) was introduced for lung cancer detection. The 3D CNN is a highly predictive architecture with an improved steepest descent input signal that increases the appearance of tumor tissues. The LUNA database is used to assess the proposed Alex Net detection technique to an existing 2D CNN training classifier. Due to a lack of testing data, the proposed model is unsuccessful, with just 10% of the training database being utilized.

Tajbakhsh and Suzuki (26) examined the performance of CNNs and MTANNs for detecting and classifying lung nodules. Achieving 100% sensitivity and 2.7 false positives per patient, MTANN exceeds the top performing CNN (AlexNet) in their testing. The MTANNs achieved an accuracy of 0.88 in classifying nodules as benign or malignant.

Gu et al. (27) suggested a unique 3D-CNN CAD system for lung nodule detection. They used a multiscale technique to improve the system’s detection of nodules of varying sizes. The suggested CAD system considers preprocessing, which is common in standalone CAD systems. It uses volume segmentation to create ROI cubes for 3D-CNN classification. After categorization, DBSCAN was used to blend adjacent regions that could be from the same nodule. Larger scale cubes have lesser sensitivity (88%) but an average of one false positive per patient, according to the LUNA16 dataset.

The multi-section CNN model suggested by Sahu et al. (28) uses multiple view sampling to classify nodules and estimate malignancy. Their proposed model is faster than the widely utilized 3D-CNNs. To develop their system, they employed pre-trained MobileNet networks and sample slices extracted in various directions. On the LUNA2016 dataset, the suggested model had a sensitivity of 96% and an AUC of 98%. They estimate the class likelihood of malignancy using a logistic regression model. It estimated malignancy with 93.79% accuracy. Because it is so light, it can be used on smaller devices like phones and tablets.

Deep3DSCan was proposed by Bansal et al. (29). To do so, they applied a deep 3D segmentation technique on CTs. The ResNet-based model was trained using a combination of deep fine-tuned residual network and morphological features. The LUNA16 dataset was utilized for training and testing. The proposed architecture achieved an F1 score of 0.88 in segmentation and classification tasks.

In Jothi et al. (30), the framework designed a controlled CNN classifier for patients with lung cancer to detect potential adenocarcinoma (ADC) and squamous cell carcinoma (SCC). CNN has already been verified using authentic Non-SCLC patient information from preliminary phase afflicted subjects collected at Massachusetts General Hospital (31). In the record, there are 311 data phases that have been collected. The created CNN, which is a VGG system training predictor, only had a 71% AUC predictive performance, which was insufficient. The VGG CNN model’s flaw is that it hasn’t been preprocessed for background subtraction or image reconstruction fragmentation, which increases the predictive accuracy. In Kasinathan and Jayakumar (32), the new cloud-based tumor recognition model was developed. The author analyzed various standard dataset “CT-scans and PET-scans” for segmenting the ROC and for recognizing the tumor. In Jakimovski and Davcev (33), the framework proposes a novel deep learning method based on binary particle swarm optimization with a decision tree (BPSO-DT) and CNN to identify the malignant or normal cells in the lung region using the genetic features (34).

## 3 Proposed Methodology

### 3.1 System Overview

**Figure 2:**
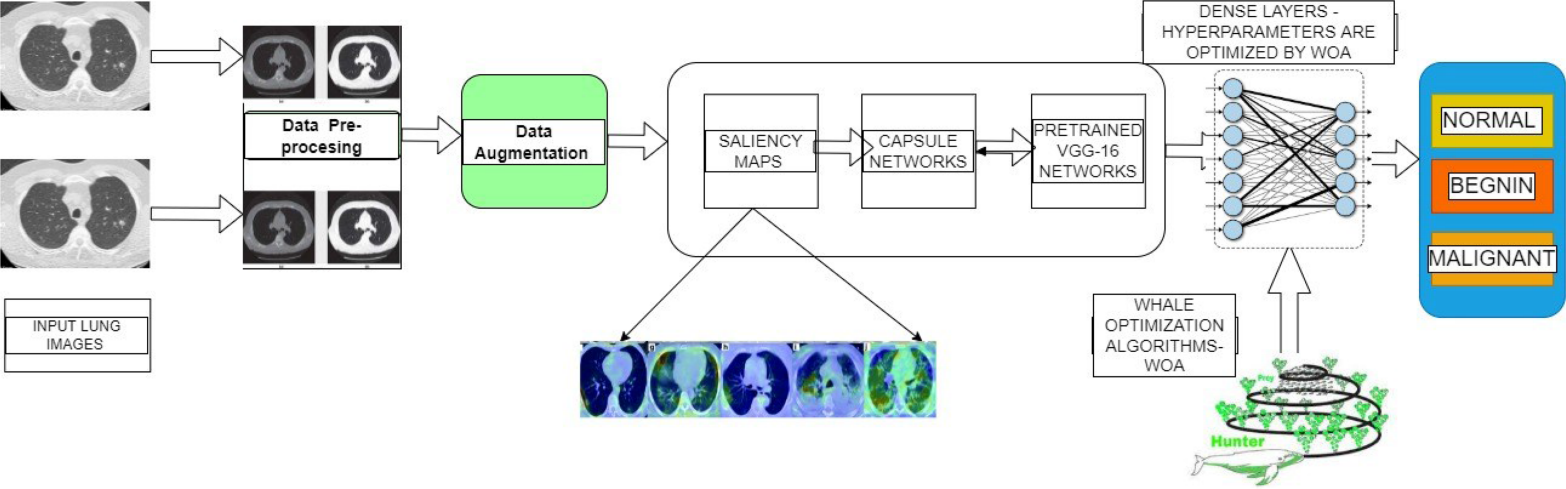
Overall Working Flow Diagram for the Proposed Architectures.

**Figure 2** shows the complete architecture for the proposed framework. The working mechanism of the proposed deep learning-based diagnosis and classification system is subdivided into three important phases. Image preprocessing and augmentation process, capsule based saliency segmentation, accurate feature extraction using the pretrained transfer learning, and finally trained by the whale optimized extreme learning networks.

### 3.2 Data Preprocessing and Augmentation

As the first step, CT scans are differentiated by using the Histogram Equalization (HOE) process. This pre-processing step is applied for adjusting the image intensities and contrast. The mathematical expression of HOE applied for image preprocessing is given by


(1) I=T∗N/P

Where T*N – Number of Pixels in N levels where N=0, 1, 2, 3………………255

P - Total Number of Pixels.

After preprocessing, an adaptive median filter (AMF) is applied over the images for effective denoising. AMF is a category of bilateral images which renders “clean, crisp, and artifact-free edges,” and improves the overall appearance.

**Figure 3:**
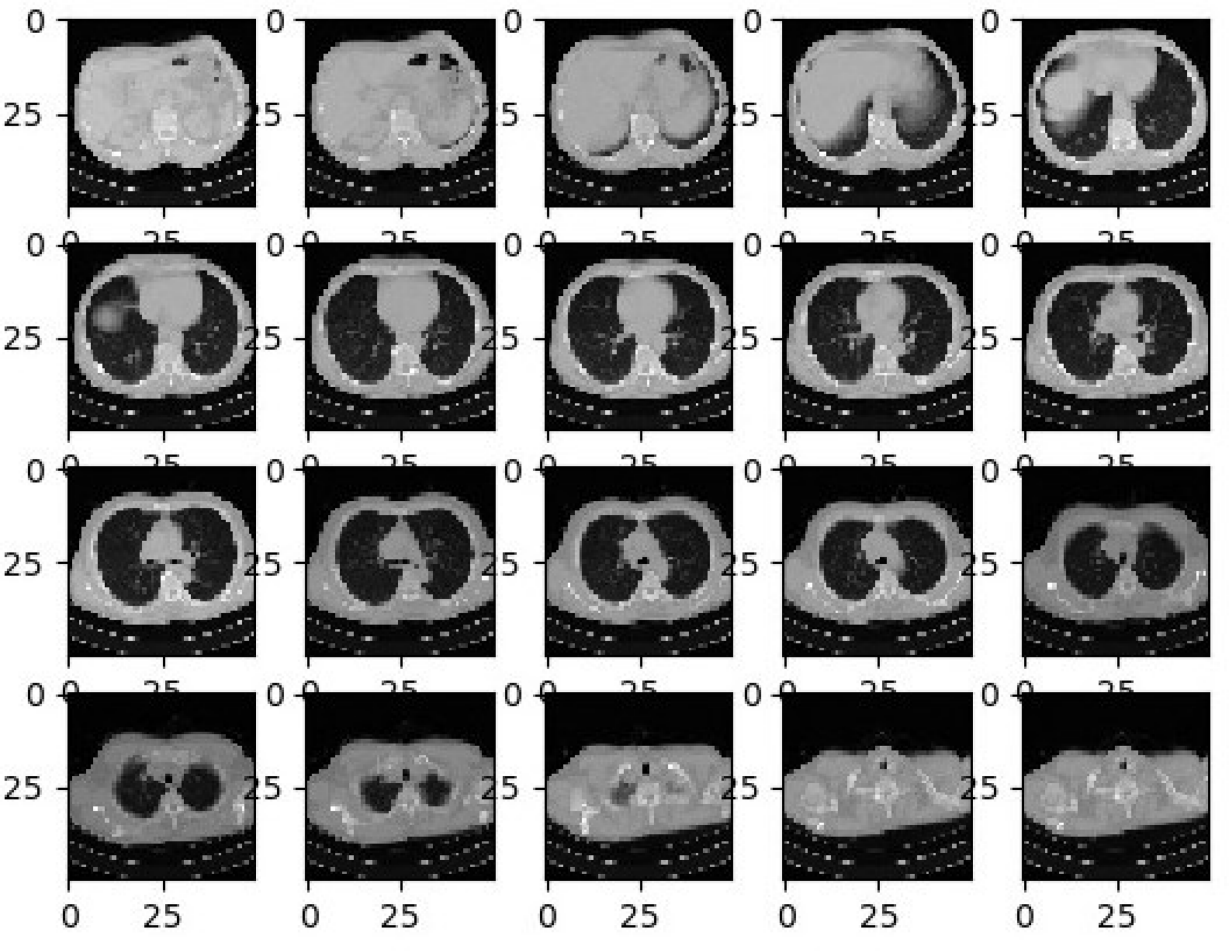
Sample CT-Lung Images after Augmentation Process.

In the second stage, the image augmentation process is used in the suggested architecture. Deep Neural Network (DNN) (35, 36) leads to overfitting problems where a limited quantity of labeled data is available. The most proficient and efficient method to tackle this problem is data augmentation. During the data augmentation phase, each image undergoes a series of transformations, producing a huge amount of newly corrected training image samples. As discussed in Pei and Hsiao (37), an affine transformation is employed for efficient data augmentation. The offline transformation techniques, such as conversion, ascending, and spins are used. Inputs are correlated with the augmentation step which is extracted before the training phase and the correlated values are utilized to avoid the over-fitting issue. **Figure 3** shows the different lung images obtained after applying the offline transformations.

### 3.3 Capsule Saliency Segmentation

Segmentation is a technique of partitioning the images with different magnitudes of patterns and pixels. For segregating the images, various techniques have been established. Capsule saliency maps are a structured technique that has been presented here. It subdivides the images into compacted and diverse parts. There will be a reduction in the number of unnecessary elements in the images.

To build the saliency models (38), color difference and spatial difference is applied in a pixel-based processing in which each pixel is represented as a block. To achieve this, pixels ‘X’ of images and then disintegrated into non-overlapping blocks with size nxn where n=8 and 16, respectively. Hence the saliency maps S(k) are calculated by using the mathematical expressions given by


(2) $$S\left(k\right)=\sum_{n=8,16}X\left(n\right)*S`\left(k\right)$$

Since the location, dimensions, and shape of cancer cells are the same in their adjacent slices, the finishing saliency maps are calculated as the biased sum of the authentic (S(m), preceding (S(m1) and next blocks(S(m2)) color and spatial saliency as mentioned in Banerjee et al. (38)


(3) S(m)=w1∗S(m1)+w2∗S(m)+w∗S(m2)

After calculating the saliency maps, post-processing techniques need to be adopted for refinement of segmentation images. Active contour methods [28] are used for the recognition of cancer cells in the most consecutive twin blocks. Also, accurate separation of cancer cells from the other parts of CT scan images is badly needed to give a precise output. Moreover, active contours are based on image intensity, which probably fails in differentiating the cancer cells. Additionally, these contour methods require higher computation time, which is considered to be a serious problem in handling larger datasets.

Motivated by this drawback, this paper introduces capsule networks with pretrained optimized models to obtain high performance and accurate detection of CT lung cancer images. Its main disadvantage seems to be that, in order to get such high standard findings, these techniques necessitate substantial fine-tuning and optimization that is clearly not feasible with massive datasets and has an impact on the recognition rates. But in this proposed system, training effort take reduced time and increase the efficiency and performance of the system.

### 3.4 Capsule Networks – An Overview

**Figure 4:**
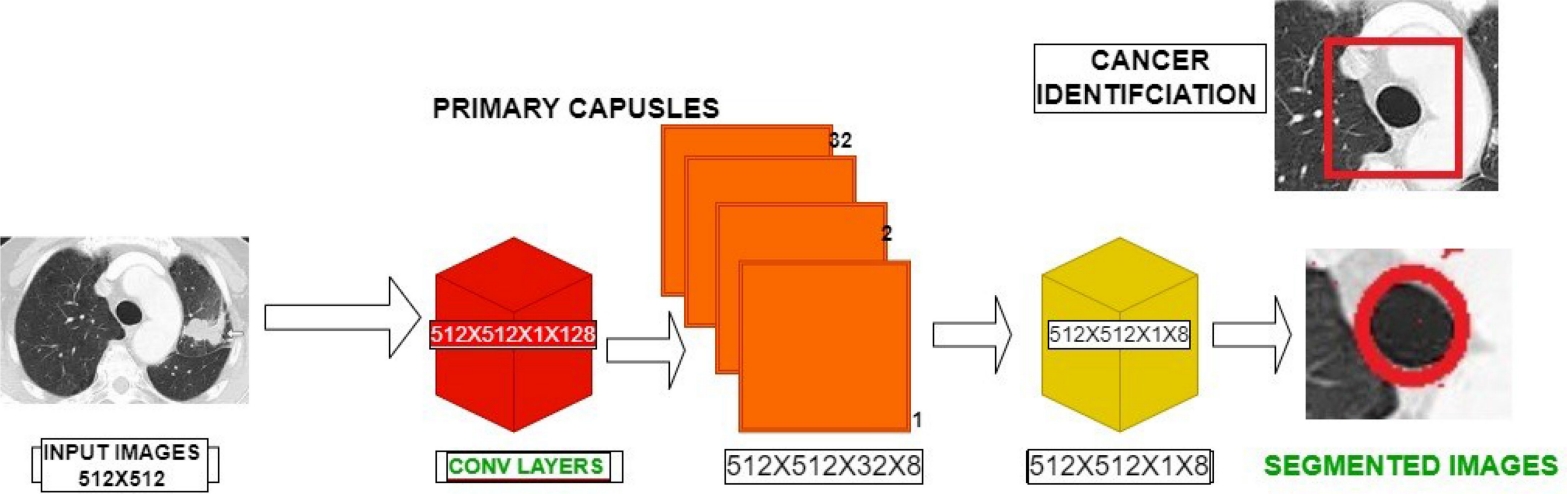
Capsule Architecture for the Saliency Based Segmentation.

Capsule network (39) is the new and upcoming network that is replacing the prevailing models. The capsule network contains four layers: 1) convolutional layer, 2) hidden layer, 3) PrimaryCaps layer, and 4) DigitCaps layer. **Figure 4** shows the entire working structure for the given training model. The capsule networks provide more advantages in categorizing the distinct saliency maps in the images. The input preprocessed visual image is given as the input to the proposed capsule networks. Capsules are groupings of cells that encrypt the location data as well as the likelihood of an object being present in an image. In capsules networking, there is a shell for every object in an image that gives:

1. Probability of existence in entities
2. Entities’ instantiation parameters

The combination of the matrices of the input variables with the weight matrix is computed to represent the essential spatial correlation between poor and large-scale features within the image.


(4) $$Y\left(i,j\right)=W_{i,j}U\left(i,j\right)*S_{j}$$

Equation (5) estimates the total weight which is calculated to determine the updating of the current capsule values and the same id feedforward into the next level of capsule determination.


(5) $$S\left(j\right)=\sum_{j}Y\left(i,j\right)*D\left(j\right)$$

At last, the squash task is used to apply non-linearity. The squashing utility translates a vector to an extreme length of one and the least length of zero while keeping its orientation.


(6) G(j)=squash(S(j))

### 3.5 Segmentation Process

**Figure 4** shows the capsule architecture employed for the saliency segmentation. The preprocessed images are encoded using the equation (2) which involves the convolutional layers and capsule layers. The convolutional operations are first performed over the preprocessed images from conv layers to the capsules of the first capsule layers often followed by the higher capsule layers. The data transformations between the one capsule to other capsule layers are formulated by mathematical equations (3) and (4). Finally, the last layer produces the saliency segmented information which are used toward the categorization.

### 3.6 Transfer Learning for Feature Extractor

In this process, the transfer learning technique is adopted for better feature extraction and classification. Transfer learning approaches are considered as the pretrained convolutional neural networks that can be repurposed to solve different image classification problems. In this research, the Inception V3 module has been implemented due to its high accuracy and high flexibility. The custom Inception-V3 weights are pre-trained using ImageNet and it considers the reshaped size of 150×150×3 for all images.

### 3.7 Classification Layers

After the segmentation process, features extracted are then fed for training the networks. In the proposed architecture, traditional training networks are replaced with feedforward networks that are based on the principle of ELM. ELM is a category of neural network proposed by G.B. Huang (40). This kind of neural network utilizes the single hidden layers in which the hidden layers don’t require the tuning mandatorily. Compared with the other learning algorithms such as “support vector machine (SVM) and Random Forest (RF), ELM exhibits the better performance,” high speed, and less computational overhead.

The working procedure of single-layer network is illustrated with the mathematical formulation which is given below. Generally, the ML classifiers or predictors follow the feature extraction, weights formulation, and identifying the final score for the given problem. The algorithm itself generates the weights and bias factors for identifying the best final score without any backpropagation or stochastic gradient approach which minimizes the computation complexity. This is a major benefit of ELM compared to other networks (41). Due to this, ELM reduces the training error that achieves better results. Most of the categorization problem utilizes this single-layer network and many applications adopt this network for low-level data availability.

In the below statistical estimation, the extracted features are represented as “p” points with their objective function (i.e., sigmoid) where the final score is denoted as a linear graph. The concealed layer may include N-number of nodes which is not tuned mandatorily. The concealed layer’s weights are assigned at random (counting the biàs loads). Nodes are not irrelevant; however, they do not need to be calibrated, and the concealed synapse characteristics might be created arbitrarily even in advance. That is, before dealing with the data from the training set. The system yield for a single-hidden layer ELM is given by equation (7)


(7) $$\omega_{s}\left(p\right)=\sum_{i=1}^{s}\alpha_{i}ab_{i}\left(p\right)=ab\left(p\right)\mu$$

where, p→ input

“L” denotes the output weight vector and it is denoted as


(8) $$\mu ={\left[\mu_{1},\mu_{2},\text{.......},\mu_{s}\right]}^{T}$$

(9) $$ab\left(p\right)=\left[ab_{1}\left(p\right),ab_{2}\left(p\right),\text{.........},ab_{s}\left(p\right)\right]$$

(10) $$ab=\left[\begin{array}{c} ab(p_{1})\\ ab\left(p_{2}\right)\\ .\\ .\\ .\\ ab\left(p_{s}\right) \end{array}\right]$$

The minimal non-linear least square method is used to denote the basic calculation of ELM that is represented by the below equation.


(11) $$ab`=ab*L=ab^{T}{\left(ab*ab^{T}\right)}^{-1}L$$

Where ab* → inverse of “ab”: Moore-Penrose generalized inverse.


(12) $$ab`=ab^{T}{\left(\frac{1}{D}ab*ab^{T}\right)}^{-1}L$$

Hence the output function can be found by using the above equation


(13) $$\omega_{s}\left(p\right)=ab\left(p\right)\alpha =ab\left(p\right)ab^{T}{\left(\frac{1}{D}ab*ab^{T}\right)}^{-1}L$$

Though extreme learning principle-based feedforward networks produce the best performance, non-optimal tuning of hyperparameters such as input weights, hidden neurons, and learning rate affects the accuracy of classification. Hence, optimization is required for tuning the hyperparameters for achieving the best performance. The next section discusses the proposed algorithm used for optimization of the extreme learning networks.

### 3.8 Optimized Extreme Learning Models

This section discusses whale optimization algorithm (WOA) and proposed optimized extreme classification layers.

#### 3.8.1 Whale Optimization Algorithms

**Figure 5:**
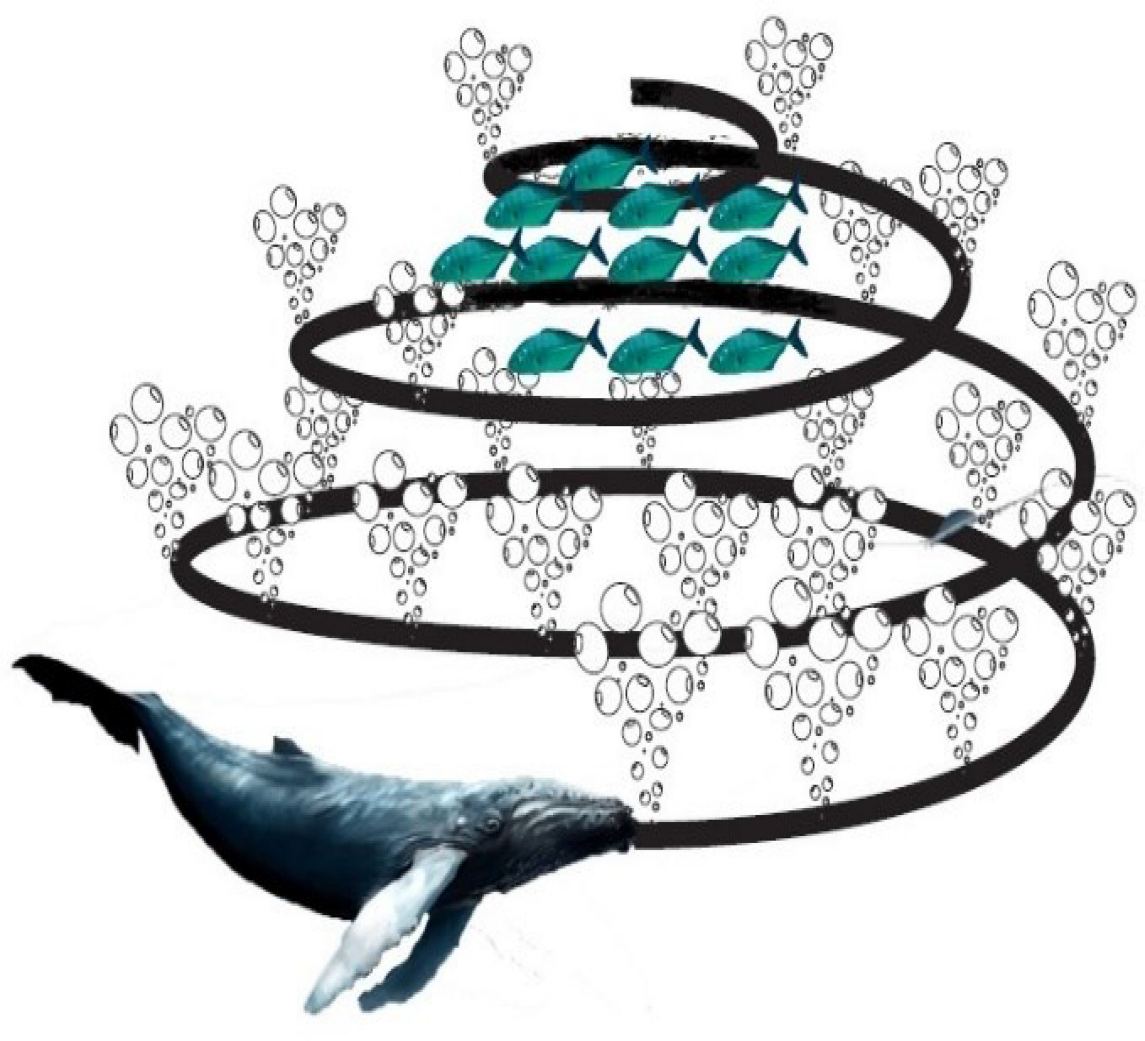
WOA Basic Structure.

WOA, first proposed in Mukherjee et al. (42), has sparked renewed interest in recent years. This stochastic search technique is computed by the following simulation of humpback whale behavior and movements in their search for food and supplies. WOA was inspired by the bubble-net attacking method, in which whales target fish by forming tailspin bubbles surrounding them down to 12 meters below the surface, subsequently swimming back up to trap and grab their prey, as shown in **Figure 5**. The search phase in this method is characterized by a randomized hunt for food based on the spatial location of whales, which can be statistically interpreted by automatically updating responses rather than picking the appropriate ones by selecting random solutions.

In addition to this intriguing behavior, WOA differs from other optimization algorithms in that it only requires the adjustment of two parameters. These variables allow for a smooth transition between the exploitation and exploration phases.

**Encircling prey:** The search process initiated from starting point and circles the food around the nearby region in order to update their process to the best target. The working process is detailed with statistical formulations.

If (c < 0.5 and mod(k) < 1)


(14) V=modu{(k.V−V.q)}

(15) V(q+1)=[V(s)−{c.q}]

where c=0.1 (constant), “V(q+1)” represent the best solution and other attributes are estimated as per the below formulations.


(16) M=mod{2∗c∗k−c}

(17) $$X_{p}=2*k$$

Where k denotes the arbitrary value within the range of {0 - 2}

**Prey Searching**: In the food searching process, the input “V” is denoted with “*V_random_*” which is estimated using the below equation.


(18) $$V=mod\left\{O.V_{rand}-V\left(q\right)\right\}$$

(19) $$V\left(q+1\right)=\left[V_{rand}\left(q\right)-\left\{P.R\right\}\right]$$

During the search phase of the WOA approach, the target was encircled and spiral upgrade was performed. Equation (19) represents the quantitative phrase for updating a new position.


(20) $$V\left(q+1\right)=R^{1}*ex^{s1}-\cos\;(2\pi p)+V^{*}\left(q\right)$$

“R” denotes the distance among the initial and updated position after each iteration and “s1” denotes the constant 0-1

#### 3.8.2 Proposed Model

As discussed, the WOA model is utilized to enhance the weights of ELM networks. In this case, the whale’s criteria for searching and fixing the prey are used as the main term to optimize the weights of ELM networks. Typically, the ELM channels are fed a randomized weight matrix and biased. The performance index is defined as the highest precision. The numerical simulations (14), (15), and (16) are used to determine input bias and weights for each repetition. These parameters are then fed into the ELM system, which generates the exponential function utilizing equations (9). If the output function equals the fitness value, the repetition will either come to a halt or continue. Whale adaptation has a slower convergence time than other meta-heuristic methods, but it takes less time to refine and improve response time. The whale optimized ELM is now used as the classification of lung cancer images. **Table 1** presents the optimized parameters used for training the network.

**Table 1:** Optimized Parameters for Whale Optimized Extreme Learning Networks.

Sl.no	Parameters	Optimized Parameters
1	No. of Epochs	100
2	Learning Rate	100%
3	No. of batches	20
4	Optimization Iterations	19
5	No. of hidden nodes	78

## 4 Proposed Framework Validation

### 4.1 Datasets Descriptions

The experiments are carried out using lung CT images which are obtained from the cancer imaging archives (https://wiki.cancerimagingarchive.net/display/Public/LIDC-IDRI). The collection contains 1018 lung CT scans from the National Cancer Institute, which were connected with proteomics and genetic experimental data. All trained radiographs are sorted into normal and cancerous tumors in this article. A benign lesion with a grade of less than 3 is called a normal nodule, while a malignancy lesion with a score of more than 3 is known as a malignant lesion. To eliminate ambiguity in lesion specimens, bronchial lesions with a value of 3 in malignancy are deleted. Separate software NBIA retriever is used for the conversion of tcia format data to DICOM image data which can be used for further processing. The detailed description of the datasets used for testing is presented in the tcia website (43).

### 4.2 Experiment Details

The whole experiment is carried out in the Intel I7CPU with 2GB NVIDIA GeForce K+10 GPU, 16GB RAM, 3.0 GHZ with 2TB HDD. The proposed architecture is implemented using Tensorflow 1.8 with Keras API. All the programs are implemented in the anaconda environment with python 3.8 programming.

### 4.3 Performance Metrics and Evaluation

The proposed architecture implements the six CNN layers for the better classification of cancer cells in lung images. **Table 2** depicts the partitioned datasets used for preparation and analysis the network.

**Table 2:** Total Number of Datasets (After Augmentation).

Sl.no	Total Number of Images	Training Data (%)	Testing Data (%)
1	78090	80	20

Various metrics such as accuracy, sensitivity, specificity, recall, and f1-score are calculated. The following are the mathematical expressions for calculating the metrics used for evaluating the proposed architecture.


(21) $$Accuracy\;=\;\frac{True\;Positive\;+\;True\;Negative}{True\;Positive\;+\;True\;Negative\;+\;False\;Positive\;+\;False\;Negative}\;$$

(22) $$Sensitivity\;or\;Recall\;=\;\frac{True\;Positive}{True\;Positive\;+\;False\;Negative}\;*100$$

(23) $$Specificity\;=\;\frac{True\;Negative}{True\;Negative\;+\;False\;Positive}$$

(24) $$Precision=\frac{True\;Negative}{True\;Positive\;+\;False\;Positive}$$

(25) $$F1-Score=2*\frac{Precision\;*\;Recall}{Precision\;+\;Recall}$$

### 4.4 Results and Discussion

This section highlights the validation results obtained through proposed tumor predictor along with other depth networks. The validation testing data have been segregated into four distinct folds (i) confusion matrix and (ii) ROC for the first iteration. In the next fold, the projected design is compared with the other prevailing transfer learning models such as convolutional neural network (CNN), Resnets-100, Resnets-150, InceptionV3, Google-Net, Mobile-Net, and Densenet-169 by computing the diverse performance metrics as mentioned in **Table 4**. The proposed algorithm is tested with the random 900Lung CT (50% benign, 50% normal, and 50% malignant) scan images in order to overcome the imbalance problems.

**Figure 6:**
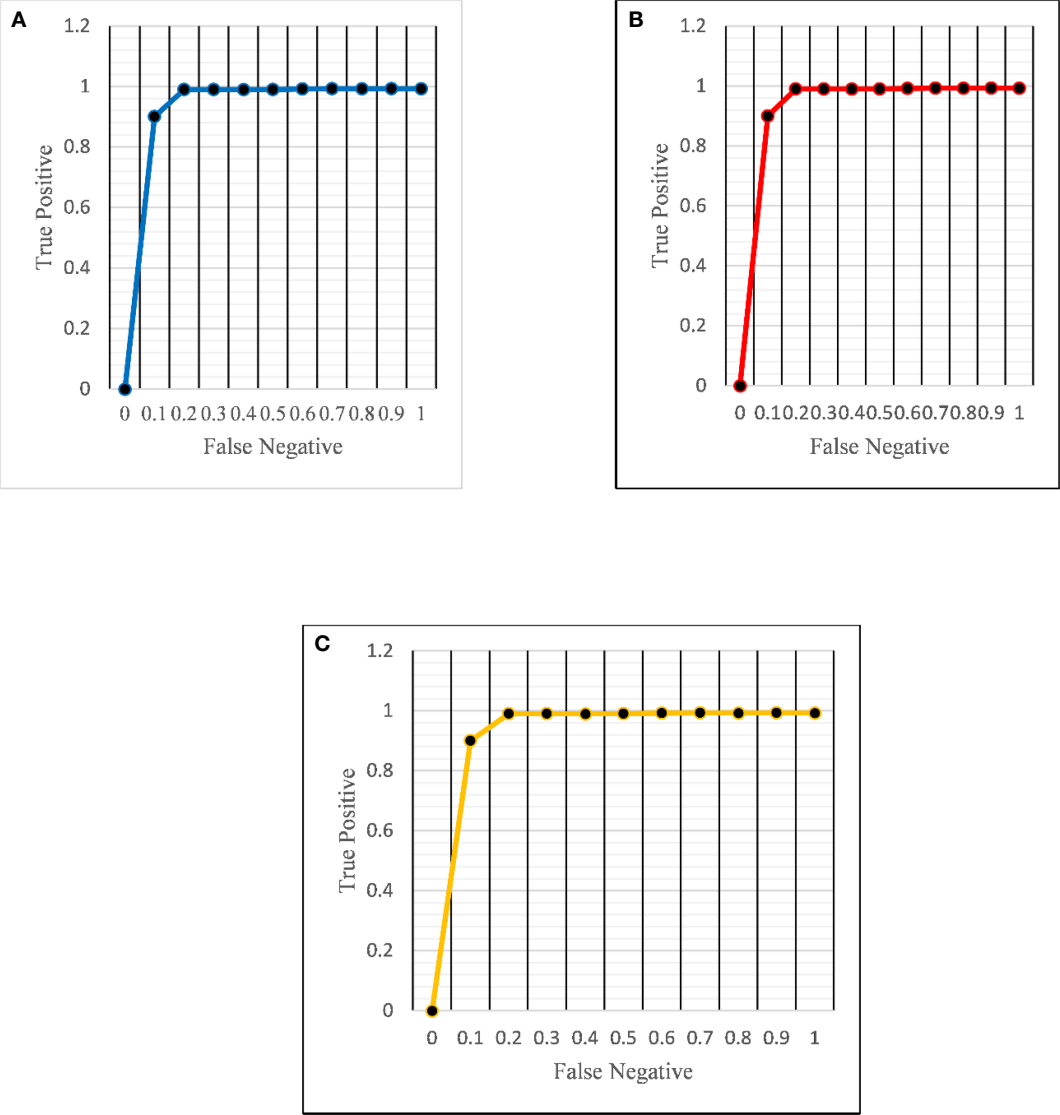
ROC curves for the proposed architecture in detecting **(A)** normal **(B)** benign and **(C)** malignant images.

**Figure 7:**
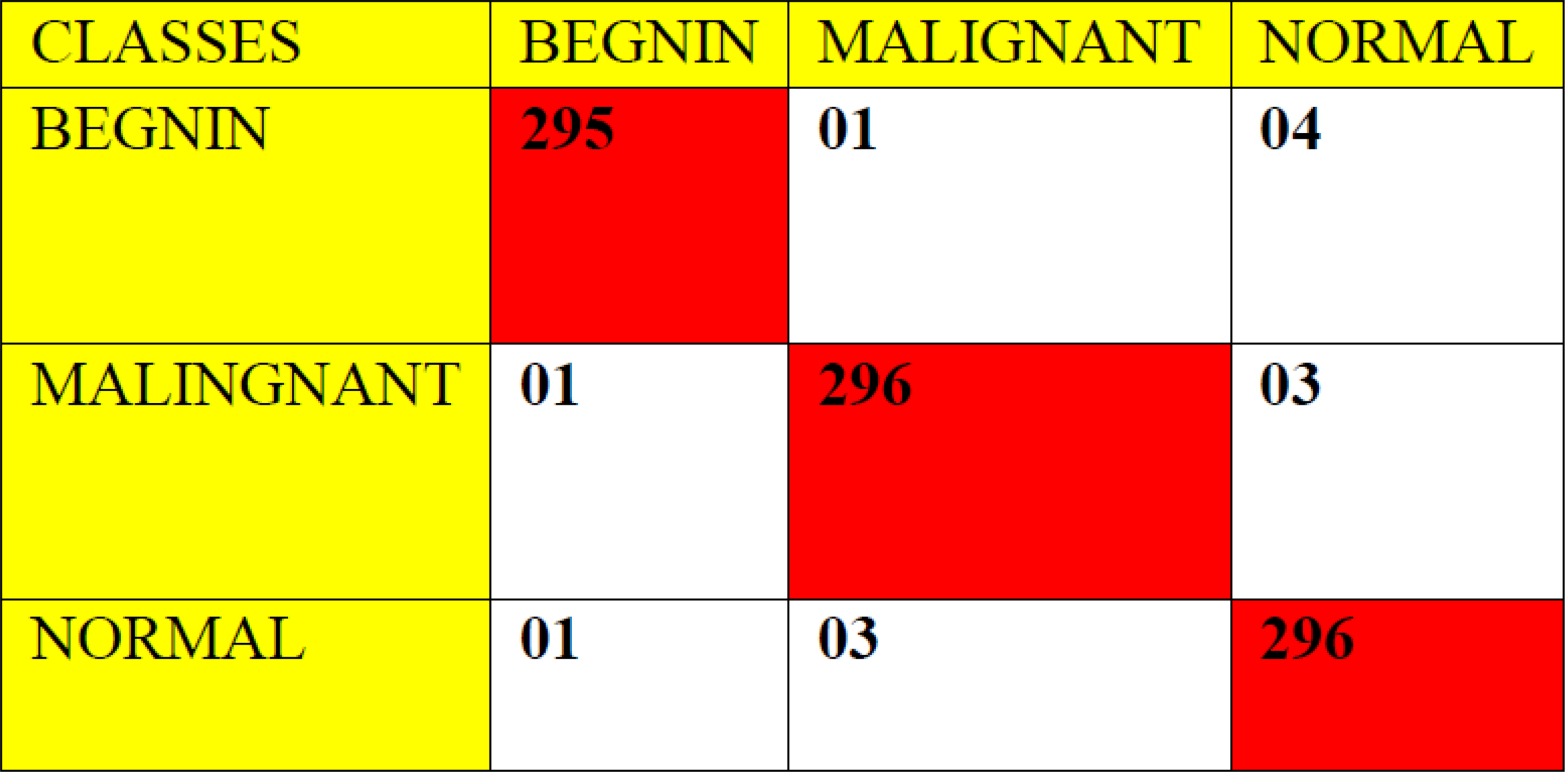
Confusion matrix for the proposed architecture using 900 random tested images.

**Figure 8:**
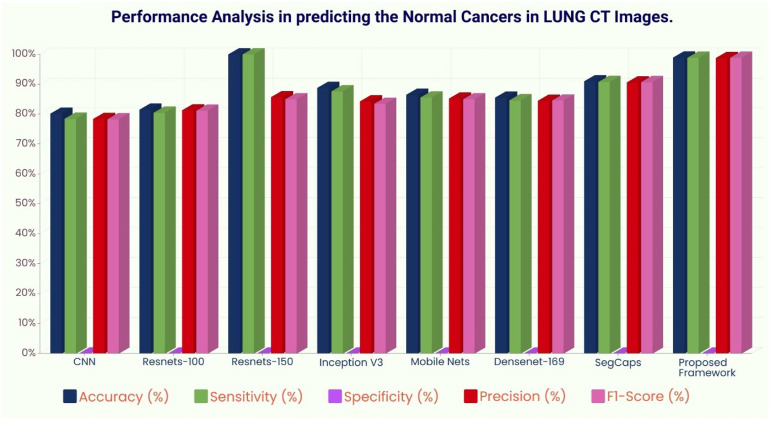
Performance analysis in predicting normal tissue in lung CT images.

**Figure 9:**
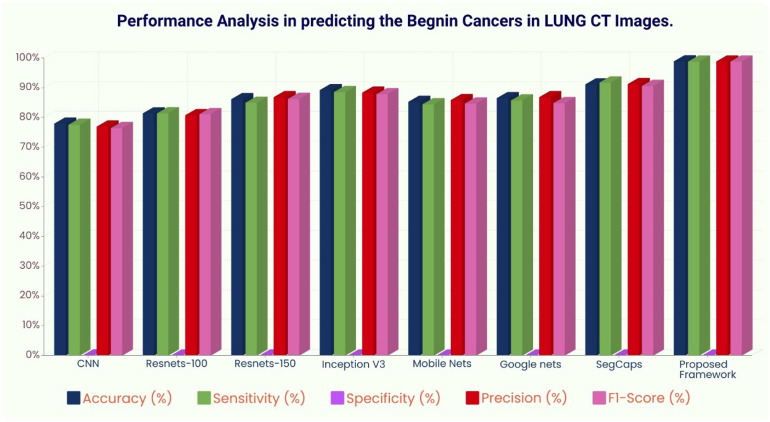
Performance analysis in predicting benign tissue in lung CT images.

**Figure 10:**
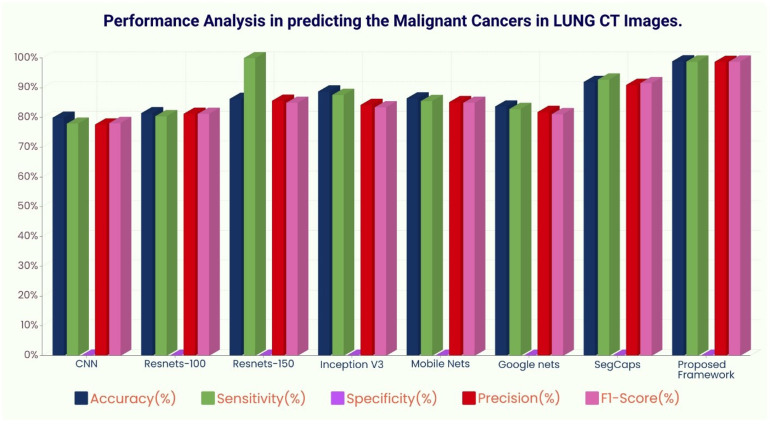
Performance analysis in predicting malignant cancer in lung CT images.

The ROC curve (**Figure 6**) and the confusion matrix (**Figure 7**) of the proposed framework in detecting the categories of CT scan lung Images. **Tables 3**–**5** highlight the performance obtained through presented framework that is associated with other prevailing algorithms. From **Table 3**, it is found that the suggested algorithm has shown the accuracy of 98.95% with 98.85% sensitivity, 98.76% precision, and high f1score of 98.85% in detecting the normal, benign, and malignant CT images. A similar performance is found in **Table 4** in detecting images of malignancy. **Tables 3**–**5** show that fusion of saliency with capsule and optimized transfer learning optimized has shown the better detection ratio using the presented network than the traditional methods. **Figures 8**–**10** represent the performance analysis in predicting the normal, benign, and malignant cancer in lung CT images.

**Table 3:** Different deep learning architectures’ performance such as accuracy, sensitivity, specificity, precision, and recall in predicting normal tissue in lung CT images.

Algorithm Details	Performance Metrics				
	Accuracy (%)	Sensitivity (%)	Specificity (%)	Precision (%)	F1-Score (%)
CNN	80.2	78.5	0.0224	78.4	78.3
Resnets-100	81.5	80.5	0.0020	81.3	81.2
Resnets-150	86,2	86.0	0.0142	85.7	85
Inception V3	88.78	87.67	0.013	84.3	83.5
Mobile Nets	86.5	85.6	0.0015	85.2	85.0
Densenet-169	85.54	84.67	0.00167	84.5	84.6
SegCaps	91.0	90.8	0.0010	90.6	90.7
Proposed Framework	98.95	98.85	0.0010	98.75	98.85

**Table 4:** Different deep learning architectures’ performance such as accuracy, sensitivity, specificity, precision, and recall in predicting benign tissue in lung CT images.

Algorithm Details	Performance Metrics				
	Accuracy (%)	Sensitivity (%)	Specificity (%)	Precision (%)	F1-Score (%)
CNN	78	77.6	0.0224	77	76.5
Resnets-100	81.44	81.45	0.0019	80.8	81.2
Resnets-150	86.21	85.0	0.0150	86.9	86.3
Inception V3	89.28	88.623	0.0127	88.4	87.9
Mobile Nets	85.32	84.5	0.00156	85.9	84.75
Google nets	86.57	85.8	0.00145	86.9	84.89
SegCaps	91.2	91.8	0.0090	91.3	90.67
Proposed Framework	98.95	98.85	0.0015	98.9	98.89

**Table 5:** Different deep learning architectures’ performance such as accuracy, sensitivity, specificity, precision, and recall in predicting malignant cancer in lung CT images.

Algorithm Details	Performance Metrics				
	Accuracy (%)	Sensitivity (%)	Specificity (%)	Precision (%)	F1-Score (%)
CNN	80	78	0.0224	77.7	78.2
Resnets-100	81.5	80.5	0.0020	81.3	81.3
Resnets-150	86.32	86.0	0.0142	85.7	85
Inception V3	88.78	87.67	0.013	84.3	83.5
Mobile Nets	86.5	85.6	0.0015	85.2	85.0
Google nets	83.784	82.9	0.0018	81.9	81.2
SegCaps	92.0	92.83	0.0080	91.0	91.6
Proposed Framework	98.95	98.85	0.0010	98.75	98.85

## 5 Conclusion

This research goal is to detect and classify malignant and benign cancer cells using CT scan lung images. To detect the location of cancer cells, this work uses the capsule-based saliency segmentation and transfer learning-based feature extraction. Furthermore, the proposed architecture employs the whale-based classification layers to achieve better accuracy. Tensorflow 1.8 tool with Keras API has been used to evaluate the presented tumor detection approach, and various performance metrics such as accuracy, precision, recall, specificity, and f1-score are calculated and analyzed. The experimental results show that the proposed architecture has achieved the best results associated with other standard architectures and obtained the best peak results. In the future, more vigorous testing is required using larger real-time clinical datasets. Additionally, the proposed algorithm needs improvisation in terms of computational complexity which will play a significant role in the analysis and identification of tumor cells as per radiologists’ perspective more accurately in future.

## Data Availability Statement

The raw data supporting the conclusions of this article will be made available by the authors, without undue reservation.

## Author Contributions

All authors listed have made a substantial, direct, and intellectual contribution to the work, and approved it for publication.

## Conflict of Interest

The authors declare that the research was conducted in the absence of any commercial or financial relationships that could be construed as a potential conflict of interest.

## Publisher’s Note

All claims expressed in this article are solely those of the authors and do not necessarily represent those of their affiliated organizations, or those of the publisher, the editors and the reviewers. Any product that may be evaluated in this article, or claim that may be made by its manufacturer, is not guaranteed or endorsed by the publisher.
