# Supplementary figures and images for: Early Prediction of Lung Cancers Using Deep Saliency Capsule and Pre-Trained Deep Learning Frameworks
Source: Front Oncol. 2022 Jun 17;12:886739. doi: 10.3389/fonc.2022.886739 (PMC9247339; doi:10.3389/fonc.2022.886739)

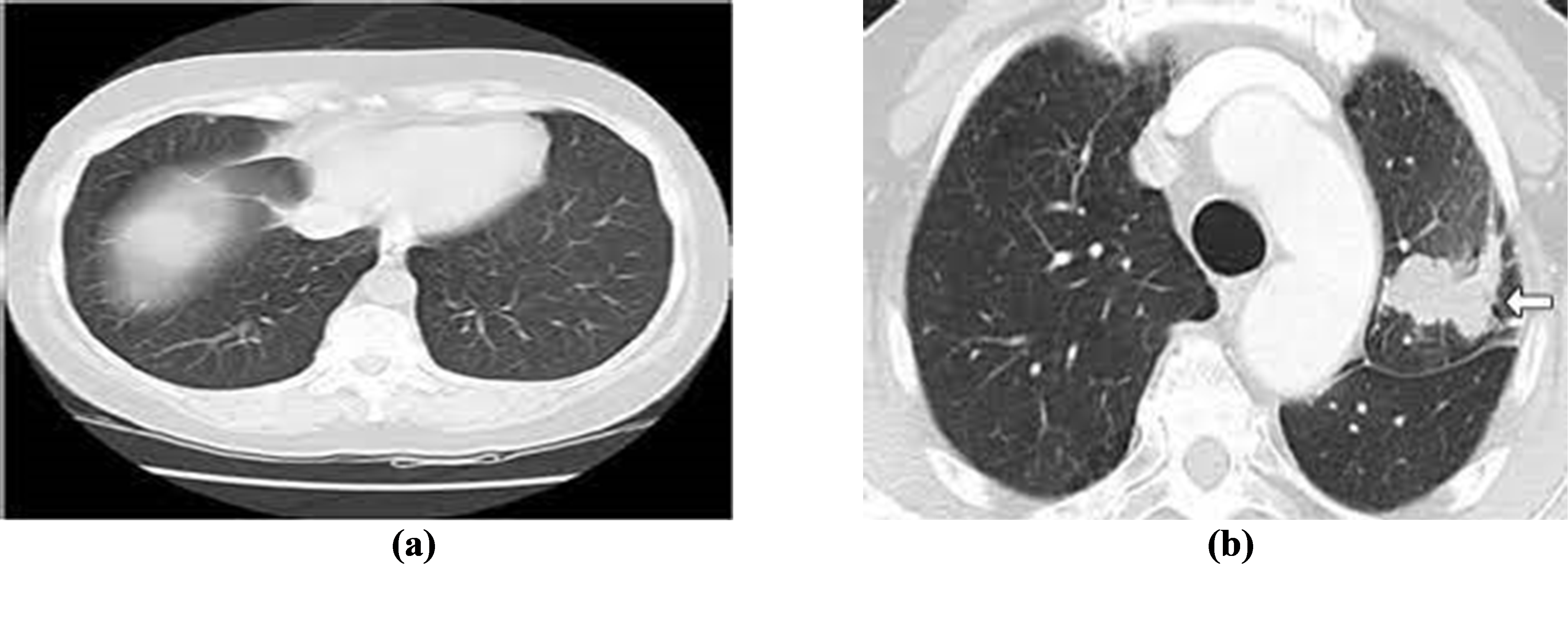

Supplement: Supplementary file 1 [file DataSheet_1.zip › 1.png]

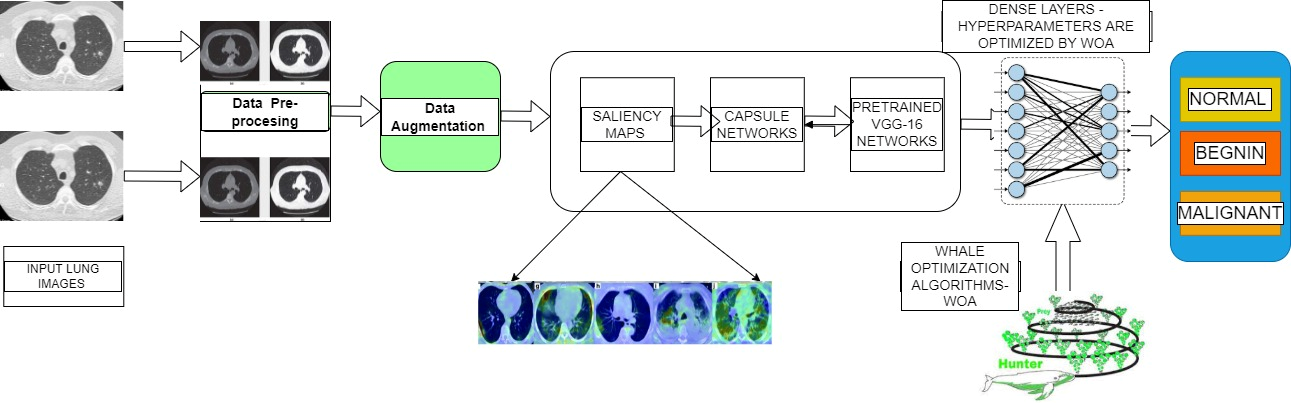

Supplement: Supplementary file 1 [file DataSheet_1.zip › 2.png]

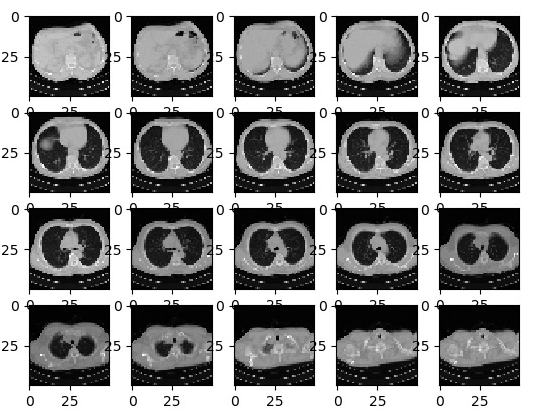

Supplement: Supplementary file 1 [file DataSheet_1.zip › 3.png]

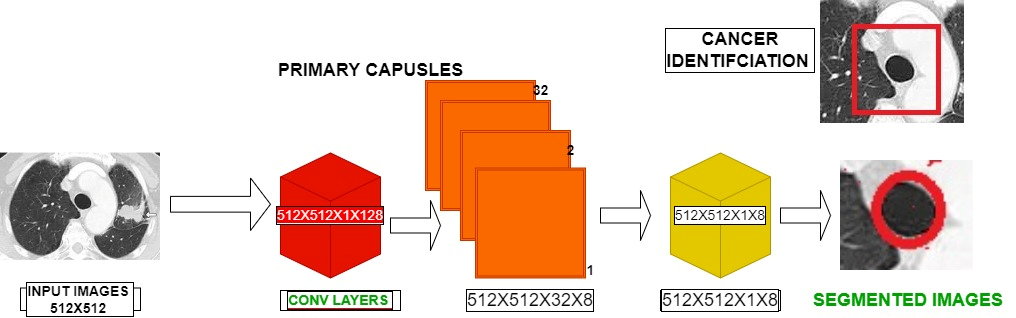

Supplement: Supplementary file 1 [file DataSheet_1.zip › 4.png]

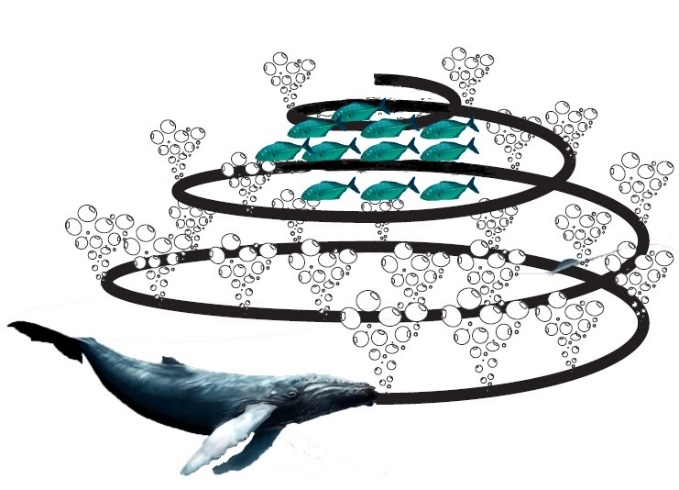

Supplement: Supplementary file 1 [file DataSheet_1.zip › 5.png]

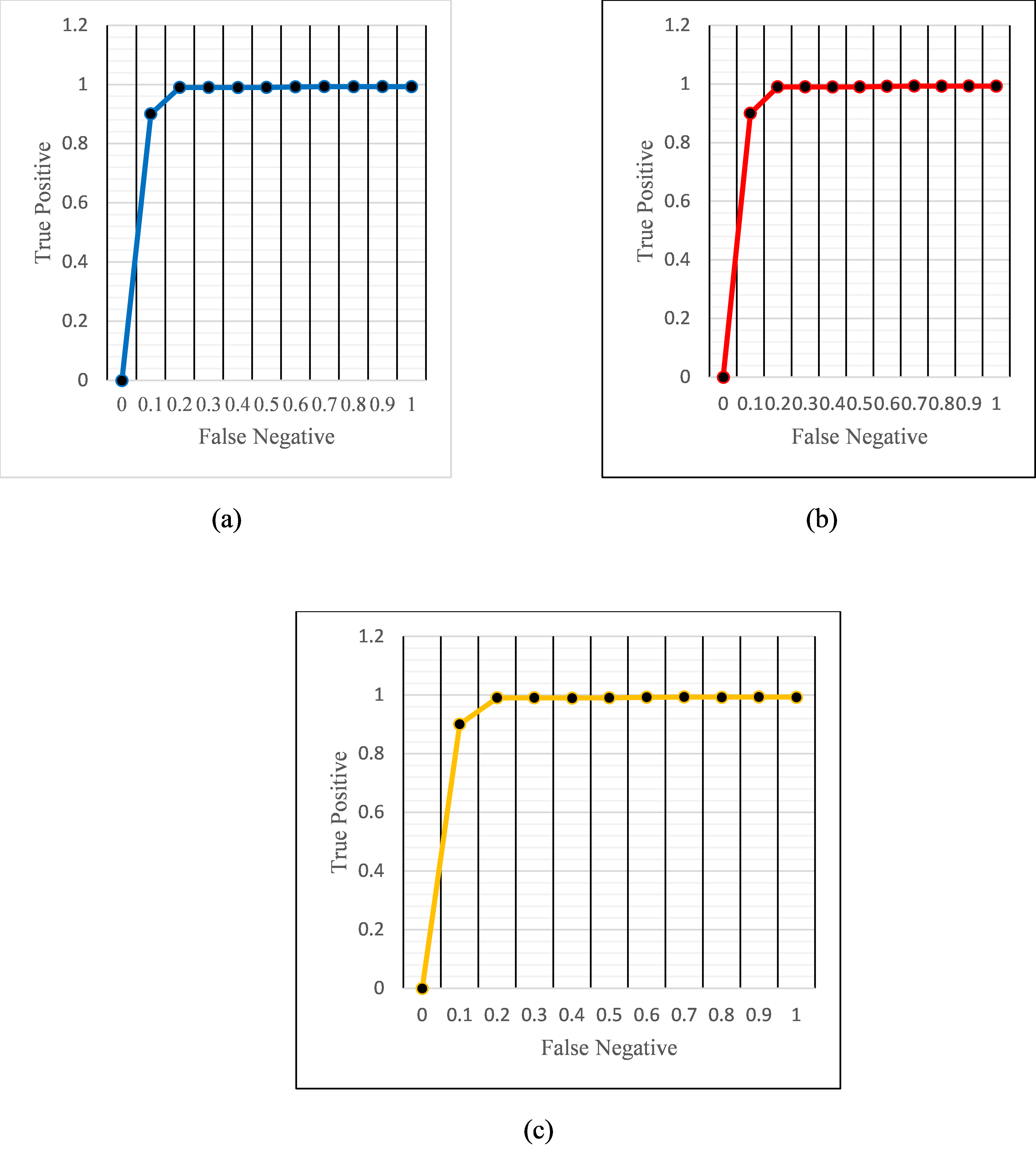

Supplement: Supplementary file 1 [file DataSheet_1.zip › 6.png]

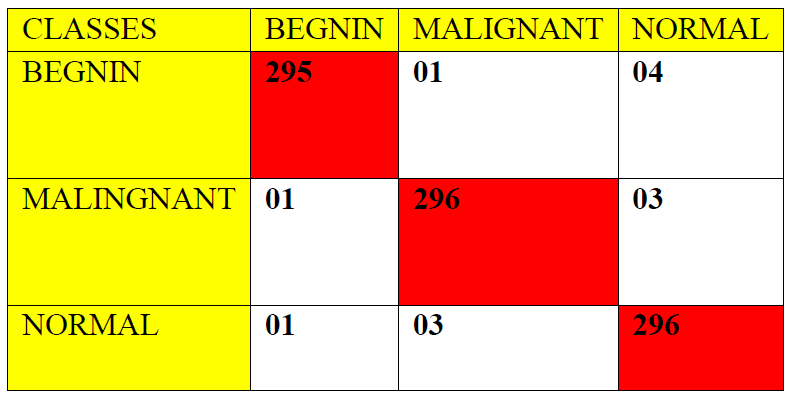

Supplement: Supplementary file 1 [file DataSheet_1.zip › 7.png]

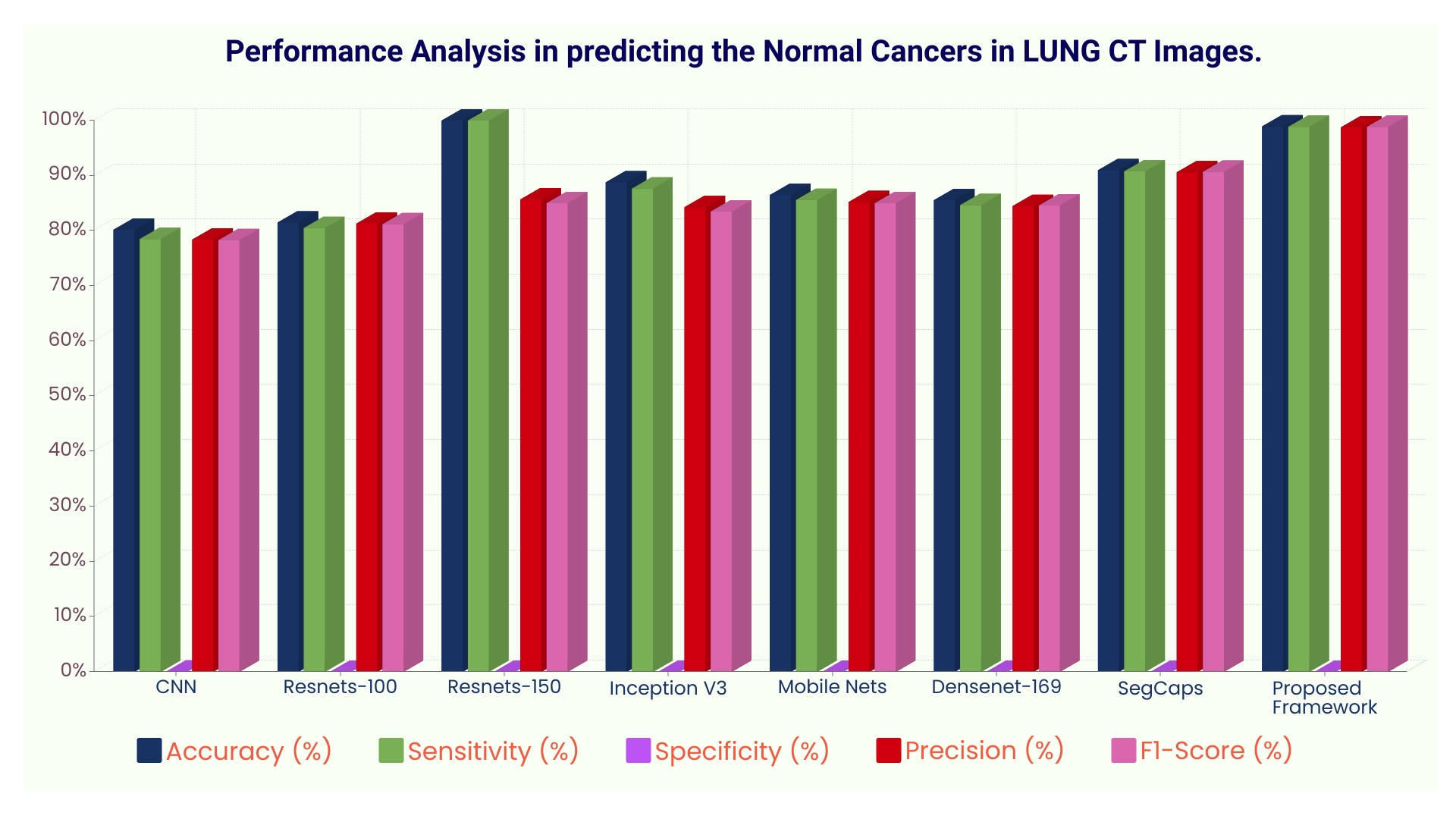

Supplement: Supplementary file 1 [file DataSheet_1.zip › 8.jpg]

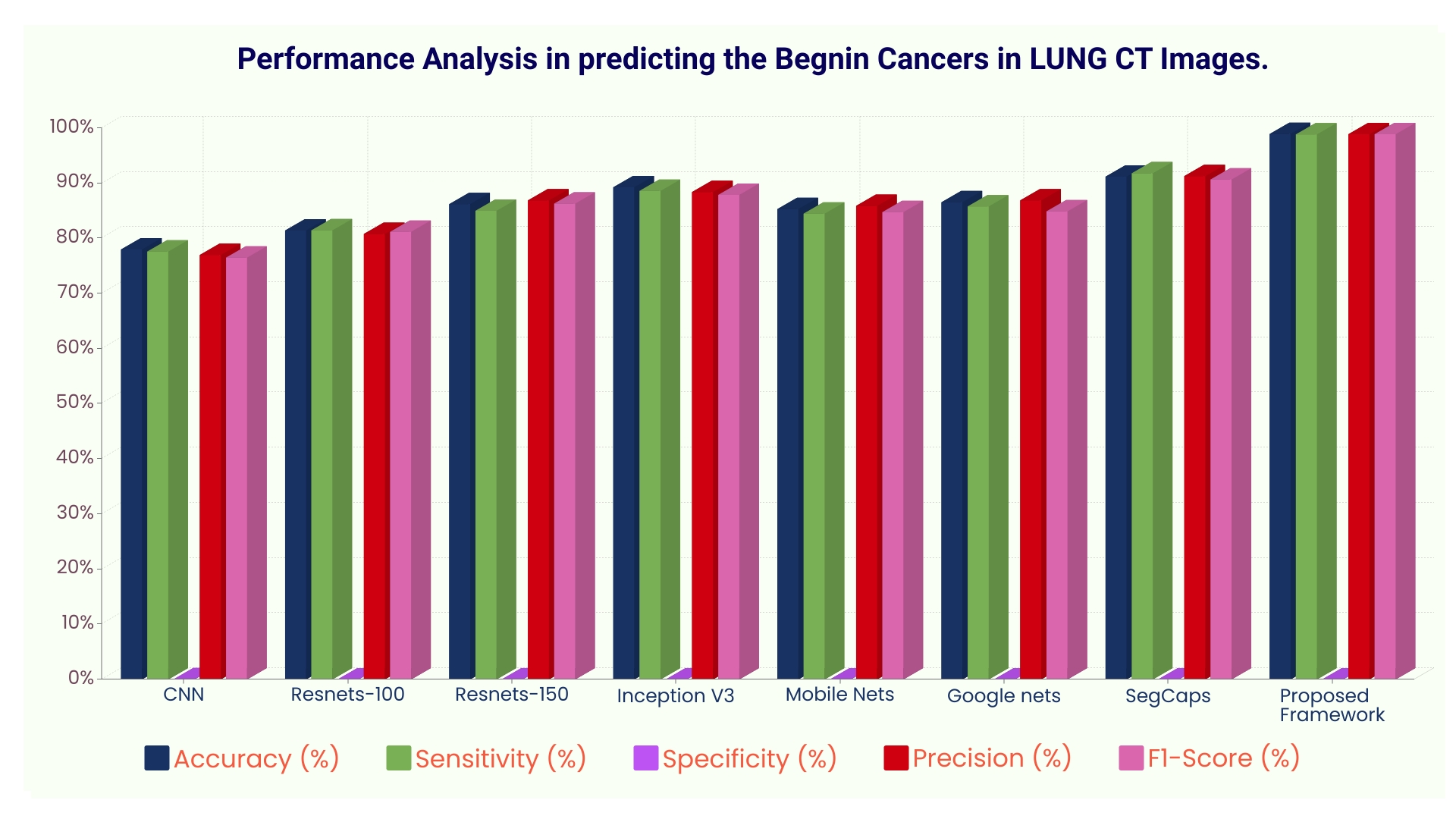

Supplement: Supplementary file 1 [file DataSheet_1.zip › 9.jpg]

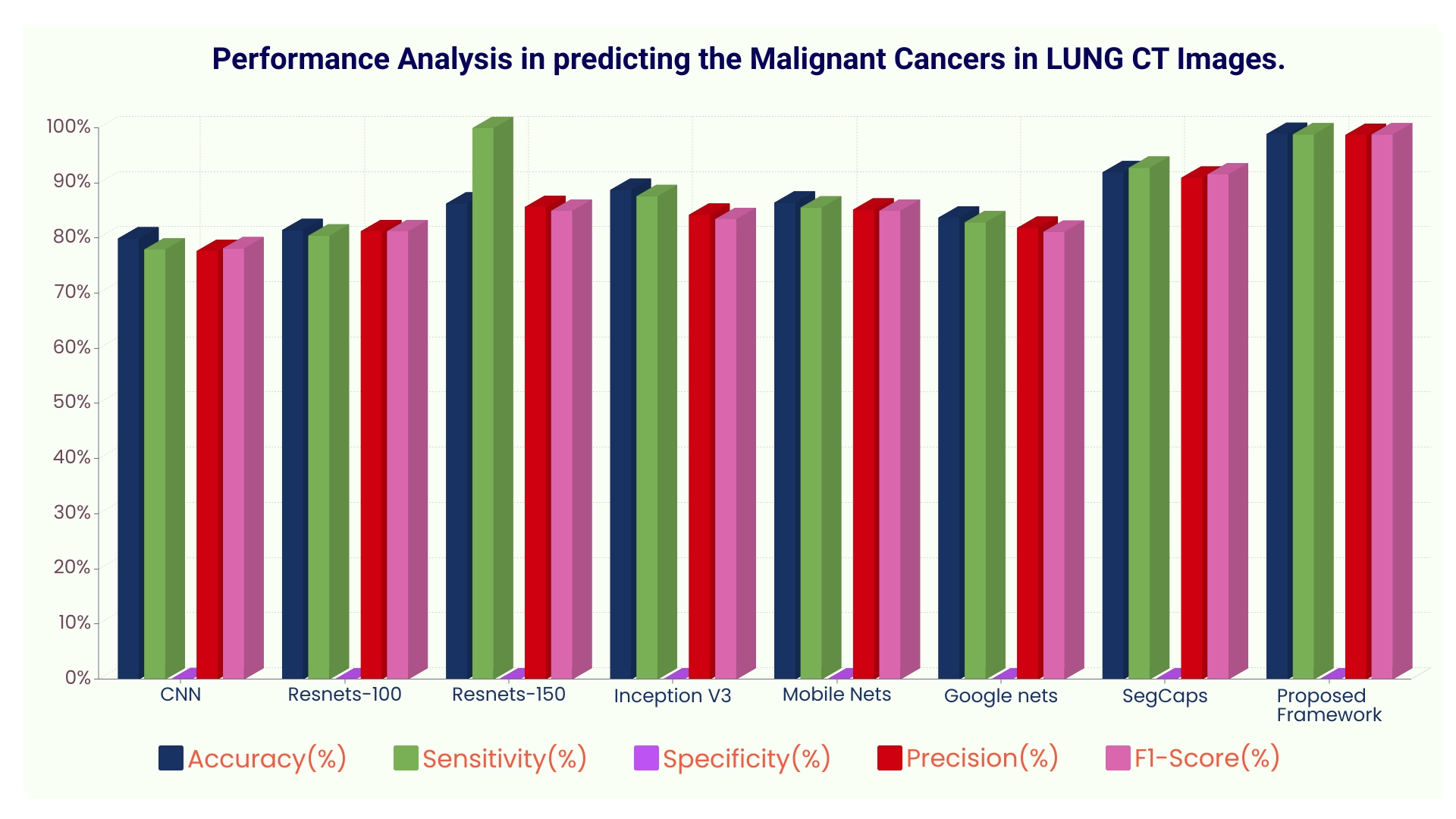

Supplement: Supplementary file 1 [file DataSheet_1.zip › 10.jpg]

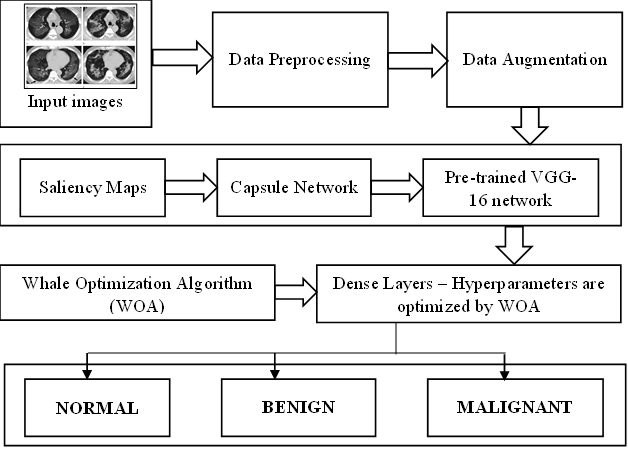

Supplement: Supplementary file 2 [file DataSheet_2.zip › 2a.png]

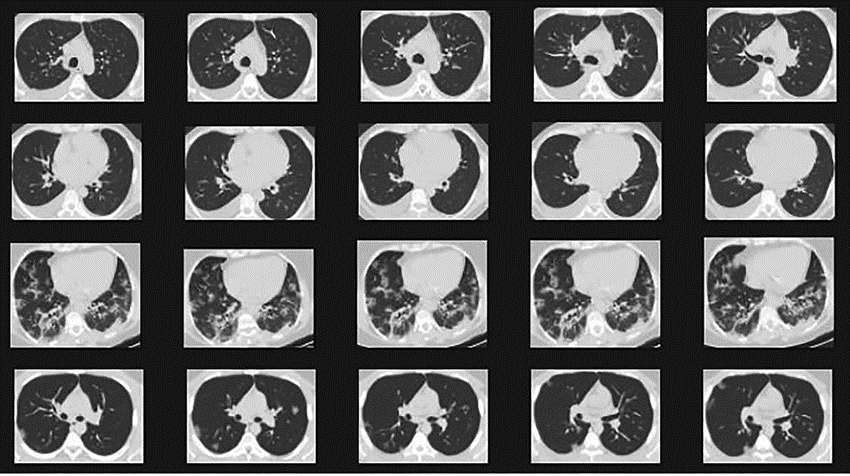

Supplement: Supplementary file 2 [file DataSheet_2.zip › 3a.png]

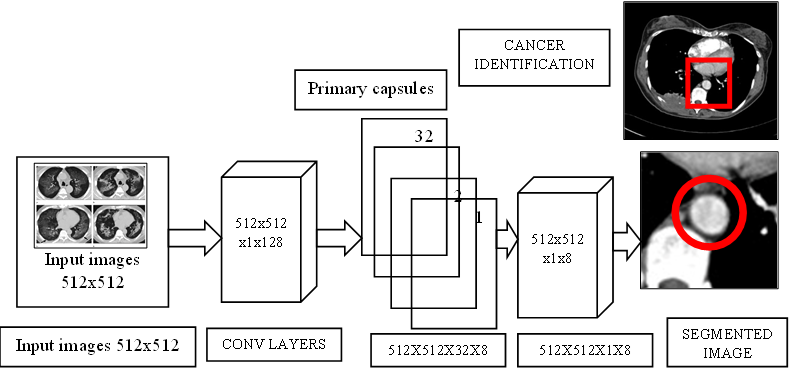

Supplement: Supplementary file 2 [file DataSheet_2.zip › 4a.png]
